# Autobiography of the Insane

**Published:** 1857-01-01

**Authors:** 


### Art. III.—AUTOBIOGRAPHY OF THE INSANE.

In continuance of our series of papers illustrative of the phenomena of insanity, as delineated by those who have suffered from the effects of alienation of mind, we reprint, with much pleasure, an interesting paper which appeared in the July number of the "*American Journal of Insanity.*" It is written by a young gentleman of talent and literary pursuits, who was a patient in the New York State Lunatic Asylum. He suffered from an attack of acute mania, attended by considerable physical prostration, following a protracted attendance upon religious exercises. The disease was of five months' duration when he was discharged recovered.

Man, the most perfect and complicated in structure of all God's workmanship, is at the same time subject to the greatest number and variety of injurious agencies. This liability is, indeed, a natural consequence of the complexity of his organization. Possessed of a composite nature, in which the material and spiritual elements are strangely interblended and harmonized, he is at once subject to the imperfections and evils incident to both. Add to this the effect of highly artificial modes of life, by which nature seems crossed and thwarted at every turn, and of unnatural habits voluntarily contracted, which add insult to her injuries, and the passage from the cradle to the grave is like running a gauntlet of perils, from which it is really wonderful that so many escape unharmed.

"The ills which flesh is heir to" may be classified under three general heads: those diseases which attack the body exclusively; those which affect the mind exclusively; those which impair the connexion between the mind and body, and hence are commonly called nervous. The first of these classes has occupied the attention of men from a very early period in the world's history, and the treatment of it belongs entirely to the science of medicine in its various branches. It is of the second class that I wish to speak.

That species of disease which attacks the mind, producing insanity in its various forms, though it has always been prevalent in the human family, and is often more dreadful in its results than any other, has, till within a comparatively recent period, received but little medical attention, probably because it has been thought incurable. The ancients considered insanity as a direct visitation from the gods, and the famed hellebore, which grew in the island of Anticyra, was supposed to be a cure for it. In the New Testament the insane are spoken of as those possessed with devils, and the miracle of casting out devils is now supposed to have been the restoring the lunatic to reason. Herman Melville, in "Typee," tells us that in the South Sea Islands lunatics are revered as a kind of inspired or sacred personages, and accordingly allowed the largest liberty. It is only in the most enlightened countries and in modern times that asylums have been founded, and systematic efforts made in the treatment of this formidable and mysterious disease. France, foremost in the pursuit of science, and at the head of all modern nations in works of public benevolence, has led the way in this also. There commenced that course of treatment now universally practised, by which such great advances have been made in the art of "ministering to a mind diseased." Instead of chains and brutal cruelty, which only serve to madden still more hopelessly the unfortunate wretch, kindness and sympathy have been substituted; and it has been found that these would often illumine, and sometimes entirely dispel, the Cimmerian night in which many a noble spirit lay enshrouded.

This was a great forward step in the management of insanity, but it was only the beginning; the business of accurately classifying and scientifically treating the various forms of mental derangement has yet to be accomplished. Its types are so numerous and peculiar, that it would be almost impossible ever to arrive at an accurate analysis of all of them. The most comprehensive classification, including all the varieties of mental imperfection and disease by which man is unfitted for the exercise of his powers as a rational being, would seem to be something thus: radical deficiency of intellect, which constitutes idiotcy; total derangement of all the faculties of the mind, by which the mental equilibrium is entirely overthrown, and the intellect, moral sentiments, passions, and appetites, are thrown into a complete chaos of elements, of which the primal chaos of the material world was but a feeble type; excessive activity or predominance of some particular faculty, sentiment, or propensity, or the entire occupation of the mind by some leading subject of thought, till the perceptive powers become distorted with regard to all objects connected with that object, while they remain correct on all others—this is insanity; disordered state of the nervous system, or the connecting medium between mind and body, which gives rise to hypochondria, optical illusion, and to which spectral appearances and ghost stories are said to owe their paternity.

All these forms of mental disease are complex in their character, or at least in their first symptoms, and require to be considered under two aspects, physical or physiological, and metaphysical. Since the causes of insanity are usually of a mixed character, and the disease itself almost always so, the treatment should be addressed both to the material and spiritual nature of the patient. This is what renders it difficult. Ordinary insanity often arises from excessive mental activity, by which the nervous energy is withdrawn from the general system and concentrated in the brain. Of the efficient causes of this species of insanity it is not necessary to speak. They are numerous, and will be found enumerated in the journals of insanity; but of the proximate causes or symptoms, want of sleep is the most common and obvious. When a man's "soul gets into his head," to the extent that he cannot sleep, he is in a bad way, and had better speedily adopt some means of driving it out again.

People with large, active brains and comparatively small vital powers are peculiarly liable to mental derangement, while, on the other hand, persons of predominant vital temperament have comparatively little to fear from it, for if there is a temporary excess of cerebral action, the heart, lungs, and stomach soon reassert their supremacy. A scrupulous attention to the laws of health, in relation to free, pure air, abundant exercise, suitable diet, cheerful employments, an abstinence from all exciting agencies, and an habitual exercise of calmness and self-control, will generally suffice, even with persons of high nervous temperament, to keep the vital powers in vigorous action, and hold the mind within the traces. A man should never become so scientific, so sentimental, or so religious, as to forget his dinner; for it is far better to vegetate, or lead a merely inert, animal life, than, like a comet, to "shoot madly from our spheres to affright the world."

With regard to the treatment of insanity, as has already been observed, it involves a course physiological and metaphysical. The body is first to be attended to, the nervous equilibrium restored, so that the patient shall eat and sleep well. When proper means are used at the commencement, while the patient is still rational enough to co-operate with the means, no doubt the symptoms might often be averted; but when the mind becomes completely disorganized, and the brain has begun to boil and seethe in good earnest, it is not easy to reduce it again by any material remedies. Narcotics and stimulants have but little effect at this stage of derangement, for the whole system seems to adapt itself readily to this new order of things; so that while the exciting causes may have long been removed, and the scathing billows of fire have retired, in some measure, within their original limits, the once stately edifice they have assailed remains a charred and desolate ruin, which no skill on the part of the apothecary can reconstruct.

The patient may eat and sleep with tolerable regularity again, while the mind is entirely unsettled. There only remains, then, a resort to the other method of treatment, and here a wide and unmapped region is laid open to the humane and skilful physician. He will here find that more depends upon his native good sense, knowledge of human nature, and natural sympathy, than upon his medical education. The forms of mental hallucination are so numerous and so subtle, that it is very difficult to unravel the tangled mass, and dissect out a single straight thread of thought, by the skilful management of which reason may be restored. There is usually some leading idea, some ruling fantasy in the mind of an insane man, which is the cause of all his trouble. This becomes, in the hands of a skilful physician, a decoy duck, by the successful management of which the whole flock may be secured; or, to use a still better figure, this *ignis fatuus*, which leads the poor benighted traveller through bog and briar, and hopelessly bewilders him in pathless solitudes, may become, when caught and guided by a kind and skilful hand, the beacon-light of his salvation, by which he may be softly guided back to the old highway of reason and happiness. It is not by flat contradiction and coercion that the deranged mind is set right: this at once provokes enmity, and the lunatic meets it with a total scepticism, which converts his best friends into liars and demons plotting his destruction.

Some one has very shrewdly remarked, that the difference between an idiot and a lunatic was simply this—that the former reasons falsely from correct premises, and the latter reasons correctly from false premises. With regard to the lunatic this is undoubtedly true in many cases. He is the most skilful of sophists; every minute and casual circumstance is turned to account in supporting his false theory; he weaves a chain of the most subtle and elaborate error, which requires the utmost gentleness and caution to untwist. He must be headed off by strategy, and led, for he cannot be driven, out of his delusion. He must be managed like Dominie Samson, in Guy Mannering, who had a soul so much above buttons that he could not be persuaded to put on a new suit of clothes; and the only means by which a change could be effected, when his old ones became too much worn, was by stealing into his room at night, while the worthy Dominie was asleep, taking away the old ones and hanging the new garments on the chair; so that when he arose and dressed himself in the morning, he incontinently put on the new breeches, without discovering the change till they were fairly buttoned, or rather not discovering it at all. Let some one correct, rational idea be substituted in the place of a false one, and that, too, without sensibly disturbing the superstructure, like putting a new sill in a building, and it often paves the way for a gradual and complete recovery. It becomes, as it were, a nucleus or centre of attraction, round which all the rest will slowly cluster in regular order, and thus a new, and sometimes more beautiful, creation emerge from the chaos. To accomplish this successfully, indirect methods are generally the best. For example, it is quite a common delusion with the insane that he is in the supernatural world; he loses all cognizance of time, and supposes eternity has commenced. In such a case there is but little use in denying this before him. He will believe you to be an emissary of Satan, sent to mislead and ruin his soul; but leave in his way a daily paper of a late date, or, if he be of a literary turn, a new book, by some favourite author, and the error will correct itself.

It would be a curious and interesting speculation to inquire a little into the pathology of insanity, with a view of arriving at a metaphysical analysis of it, so as to ascertain, if possible, in precisely what psychological change it consists. The error would probably be found not in the reflective or reasoning faculty so much as in the perceptive or seeing faculties, by which all external objects and their relations are viewed through a false medium, and distorted into unnatural shapes; hence the imagination, which draws upon the perceptive powers for its materials, becomes filled with wild and delusive images. In most cases of total insanity personal identity or consciousness is lost, or merged in the general chaos; and hence also it is, that the lunatic believes himself to be some other person—a hero, or prince, sometimes the devil, and sometimes the Deity himself. Without dipping too deeply into metaphysics, we might venture to suggest that the human mind, in a healthy state, is neither a simple unity nor a plurality, but rather a confederation of powers, and that consciousness is the quintessence or product of their combined and harmonious action; just as the government of the United States is the product of the combined governments of the several States, so that "*E pluribus unum*" would not be a less appropriate term as applied to the mind than to our country. In this consciousness we may suppose the soul resides in its normal state. The perceptive faculties are to the soul what the police is to a city—by them all passports must be *visaed*, so that in the rational mind no ideas of external things or their relations are allowed to enter which do not correspond with realities: thus truth and reason are maintained. But when insanity takes place, this harmonious confederation is broken up, and each becomes a petty sovereignty, independent within itself. A unity of action is lost, the perceptive faculties become careless, the gates are thrown open, and any gigantic fantasy may walk boldly in and usurp the seat of government! At the same time the spontaneous action of particular faculties may be unimpaired; the memory may be perfect, the moral sentiments correct, and the affections and sensibilities active; but all legitimate communication is cut off, unity is destroyed, reason is deposed, and the soul is a wreck:

> "Ever drifting, drifting, drifting
> O'er the shifting currents of the restless main."

The ideas of space and time, which are the fundamental conditions of all thought in rational minds, become confused, or wholly lost.

A few facts from my own experience may illustrate this point more clearly. The first symptom of insanity in my own case was want of sleep. I was myself conscious of this need of natural slumber as well as my friends, and tried in vain to obtain it from narcotics. The very consciousness of the fact that I needed repose, and my efforts to obtain it, only aggravated my excitement, and my brain grew every day more and more disturbed.

At last I began to imagine that the final dissolution of all things was coming on, thus transferring the tumult in my own mind to external nature. I was removed from the place where I was then residing, to be conveyed home in a carriage, a distance of some thirty or forty miles. It was on the Sabbath, in the month of October, and one of the most lovely days of "Indian summer." A golden haze overspread the earth, through which the blue peaks of the Catskills loomed softly on the southern horizon. Had I been well, I should have enjoyed the ride, for Autumn is my favourite season of the year; and as it was, the exceeding loveliness of the season stole in upon my fevered brain with something of its old effect. I imagined that it was my last look upon that earth that had once contained for me so much gladness and beauty. The rustling of the dead and dying leaves, and the smoking light that lay over all the landscape, confirmed the impression:

> "The sun's eye had a sickly glare,
> The earth with age was dim."

The houses as we passed seemed empty and desolate (which was, indeed, true, since the people were all gone to church); scarcely a living object met my eye, except a few people that were passing on foot or in carriages, and even they seemed more dead than alive; their faces wore a semi-inanimate, unearthly expression. As I gazed with weary, half-shut eye down the long valley, and across the brown woods that stretched away to the base of the distant mountains, there came into my mind, with sublime and soothing effect, and with all the force of reality, this fine sentence, which I believe to be found somewhere in Holy Writ—"And I saw all the kingdoms of the earth in a vision." The roads were smooth, the horses sped along briskly, and I believed this prophetic utterance was to be literally accomplished in my own case, and that I was thus, amid the profound stillness of universal nature, to ride over the whole earth, now fading with its last autumn. During the ride I struggled once to escape from the man who held me by his side, and displaced a bandage on my arm, where I had been recently bled. The blood flowed again copiously before it could be bound up, and this, together with the fatigue of my efforts, so exhausted me, that, when at evening we reached a small town on the banks of the river, my vital strength was nearly spent. I lay faint and weary, and gazed dimly upon the water while waiting for the ferry-boat. The bells were ringing for the evening service, and the streets were filled with people flocking to church. The full moon was rising in mild splendour over the eastern hills beyond the river, and the evening wind was just curling the water into a ripple. I thought the river was no other than the Jordan of Death, across which I was about to pass into the happy country beyond, and that the whole world was following me to judgment. While crossing, I turned my eye up the stream, and as the soft light lay upon the water, and the white sails of the sloops dotted the long vista, a sense of unutterable beauty filled my soul. When we were on the other side, and had nearly reached home, we passed through another village, where the bells were again ringing, and a stream of people passing along to church. I recognised every familiar object, but the same idea continued in my mind, and it seemed the bells were tolling, and the nations coming up to judgment. After I reached home I must have slept for some time, for when I next woke to consciousness I cannot precisely determine, but it seemed that the demons of madness were pursuing me again. I fled back into the scenes of the Jewish dispensation for repose. I found myself transferred into the early history of the world.

About this time the fall rains set in, and I supposed myself in the ark, flying through the stormy waters. I was lying in an upper room in the house of my brother-in-law, and as I looked out at the dreary weather, everything conspired to favour this delusion. The window-curtains were parted so that the space through which the light came in, was in the form of a steep lattice-roof, such as I remember in the old pictures of the ark. Here I obtained a short repose, but the pursuing fiend found me again, and drove me abroad through boundless space. Then every muscle and nerve seemed wrought to the utmost tension, and I imagined that the world was again dissolved into chaos, and that all living things had perished, but that I had found out the great secret of Nature, and through me the universe was to be reconstructed. I thought that I was the living, intelligent principle of electricity, and that I had power to call into my own person all the electric fluid in the world; and thus I was to give life again to my friends and others. My father had lately arrived, and he made a remark in my hearing which partially gave rise to this idea. He said he heard the wires of the electric telegraph ring as he passed along the road. I thought all the telegraph wires in the United States were employed in conducting the fluid into my body; and this gave me unnatural strength. I thought I was moving by some attraction towards the sun, and that there, in the opaque centre of the great luminary, I should at last find an eternal rest, and rejoin my friends and kindred. But these periods of intense excitement were followed by great nervous prostration, and then I would seem to lose again all my powers, the electric fluid was dispersed, the spirits of my friends were scattered again, and I seemed to be sinking through immeasurable depths of space, when I was just on the point of achieving immortal happiness. Again, as I had almost gathered in the scattered spirits, and the new earth was about complete, a comet struck us, and we were dashed into numerous fragments, upon which we were hurled flaming through the universe. Then there was a great battle in the sky, among hostile powers; some of my friends were upon separate fragments, and vast gulfs of fire yawned between us. I was left upon one small piece, with only two persons with me (these were two men who sat up with me through the night). A lurid light surrounded us, and these were enemies with whom my father, upon another fragment, and with a large squadron of my friends, was about to do battle for my recovery. I must have slept very little during this time, which was only a week, though it seemed to me a century.

The familiar faces of my friends, as they came into the room, would seem for a time to partially restore me to reason, and bring me back to the earth again. Then I heard sounds of harmony, and a noise of chains, and the voices of men outside the house, and I imagined they were trying to bind me to the earth, and attaching all the oxen and horses in the world to draw me back when I was endeavouring to fly away. Again, I would seem to rise in the air, and the house became a balloon, floating above the town in the gaze of assembled thousands. At last, failing to find rest for my soul, I fled still farther back into the past history of the world, for the purpose of reaching a period in the human race as remote as possible, or even anterior to the existence of men, so as to include all that had ever lived in the new creation, and thus reconcile all hostility among contending spirits. I betook myself to Grecian mythology, and became Apollo, or the sun himself, the source of all life.

When I was removed from the house to be conveyed to the Asylum, I suspected there was some design upon me, and resisted; but when I got into the carriage, and two of the gentlemen who accompanied me sat with me, while the third mounted the box and drove, I thought he was Phaëton, driving the horses of the sun, and that I ought to be doing it myself; and then the men by my side kept saying to me, "Never mind, sit still; he don't know the team, he don't understand the horses." Whether anything of this kind was actually said I know not, but it confirmed my impression; and though I felt personally secure from harm, I feared he would destroy himself, and produce universal ruin again, by driving my coursers. When we drove up to the Asylum, its imposing front made quite an impression upon me. I had some idea of the true character of the building, but the predominant fancy overruled it, and the building became the temple of Apollo, into the possession of which I was about to enter, as my rightful residence.

Then followed a period of unconsciousness, broken here and there only by impressions vivid enough to be recalled to memory. Heathen mythology became mixed with modern astronomy, and I was transferred from Apollo to Mars, and became the god of war. At this time I was very violent, and struggled fiercely with my attendants ; finally, getting no repose, and finding that I saw my friends no more, I despaired of getting back again, and thought myself a comet—the living intelligent head of a comet—flying through space with inconceivable velocity, and passing far beyond the confines of the habitable universe, thus leaving my friends hopelessly behind me. I lost all sense of time and space. A whizzing and careering through trackless solitudes, a sense of rapid and lonely motion at an incalculable rate, and a sinking of the heart in utter despair, are all I can recollect. But at length I began to notice the succession of day and night, and observe things about me ; then, to be sensible of hunger and thirst and clothing. This checked my career, and I now believed my friends, with the other inhabitants of the earth, were in the planet Jupiter, and that a cable had been passed over to me, by which I was moored alongside, or rather, held attached, though still at a great distance. Along this rope they passed me food and drink and clean clothes, and the spirits of my nearest friends came across, and entered the bodies of those whom I saw around me. One of the attendants I took to be my brother, though he resembled him but slightly ; another was an intimate friend, while another was my implacable enemy.

I began gradually to realize my situation—to feel that I was confined within stone walls. I tried to escape from the window, and should have precipitated myself boldly from any height, for I had no doubt whatever that I should fly direct to Jupiter, could I get into free air. An ethereal lightness seemed to pervade my whole frame, and the great stone edifice itself appeared to be sustained in mid-air. It was a long time after I began to recover and walked out before the earth seemed firm and resisting under my feet. During the day I enjoyed myself tolerably well, while I was permitted to walk the hall ; and the sight of the sun, when he occasionally appeared during the cloudy days of mid-winter, rejoiced me greatly ; but at the approach of night I fancied that I was falling into the power of evil again, and the lighting of the gas was very obnoxious to me. I tried to blow out the light, and once pulled down one of the gas-pipes, supposing that thereby I could hide the darkness and restore the dominion of the sun again. At last—

> "All these sharp fancies by down-lapsing thought
> Streamed onwards, lost their edges and did creep,
> Rolled on each other, rounded, smoothed, and brought
> Into the gulfs of sleep."

From the time I began to sleep soundly, my recovery was sure. But every night I visited Jupiter, and had entrancing visions of loveliness spread before me. I could see the convexity of the planet rising slowly before me, but yet swaying to and fro as if in uncertain equilibrium; and heaving and tossing like a balloon, or a ship at sea. From this delightful abode, I was invariably driven by my pursuing demon, and brought back to my prison again, notwithstanding the superhuman efforts of my friends to save me. About this time the news of the death of Daniel Webster, and the result of the presidential election, in which I had been considerably interested, began to make some impression upon me. At length, one day, I happened to see a new book by Ik. Marvel, and a January number of the *Opal*, and this established a correct idea of time. Then I enquired the day of the month, and began to keep that, as also the days of the week. Still there was a vast chasm behind me, and I thought I had been here millions of years. I was astonished to find, upon inquiry, that it had been but little more than two months. From this time forth I recovered rapidly. My delusive fancies broke up, and began to recede from my mind like the figures in a dissolving view. I adopted the State Lunatic Asylum as a fixed fact, and began to accommodate myself to my situation.

Such are some of the facts in my own experience of insanity. It will be seen from this, that the first step towards recovery is to correct the perceptions, so as to make things seem what they are, or what they seem to rational people—in nautical phrase, to take an observation, ascertain bearings and distances, and write up the log. After once recovering the ideas of time and space, and firmly fixing them, consciousness will come back to its original seat, and adapt itself again to realities. Thus the great material universe will finally swing round again to the senses, and the old order become re-established. Sometimes a sudden surprise, such as the appearance of a long-absent friend, the news of the death of a beloved one, or some other remarkable occurrence, will accomplish this at once, and restore reason instantaneously. In such cases there seems to be a powerful reaction, as if the mind were jerked back into its socket, like a dislocated shoulder-blade. I have no doubt the sudden appearance of valued friends, a few weeks after I was brought here, would have had this effect upon me.

When public benevolence reaches such a height, or the means of patients are so ample, as to induce the medical faculty to investigate the subject more thoroughly, so that scientific principles can be more generally carried into effect in the treatment of insanity, much greater success may be looked for, and, doubtless, many cases now regarded hopeless would be found not incurable.

